# Face-induced gamma oscillations and event-related potentials in patients with epilepsy: an intracranial EEG study

**DOI:** 10.1186/s12868-022-00715-x

**Published:** 2022-06-13

**Authors:** Ji-Won Kim, Katja E. Brückner, Celina Badenius, Wolfgang Hamel, Miriam Schaper, Michel Le Van Quyen, Elisa K. El-Allawy-Zielke, Stefan R. G. Stodieck, Jonas M. Hebel, Michael Lanz

**Affiliations:** 1 Department of Neurosurgery, University Medical Center Hamburg-Eppendorf, Hamburg, Germany; 2 Epilepsy Center Hamburg, Protestant Hospital Alsterdorf, Hamburg, Germany; 3 Laboratoire d’Imagerie Biomédicale (LIB), Inserm U1146 / Sorbonne Université UMCR2 / UMR7371 CNRS, Paris, France; 4 Department of Neurology, Charité-University Medicine Berlin, Berlin, Germany

**Keywords:** Temporal lobe epilepsy, Amygdala, Hippocampus, Event-related potentials, Gamma oscillations

## Abstract

**Background:**

To examine the pathological effect of a mesial temporal seizure onset zone (SOZ) on local and inter-regional response to faces in the amygdala and other structures of the temporal lobe.

**Methods:**

Intracranial EEG data was obtained from the amygdala, hippocampus, fusiform gyrus and parahippocampal gyrus of nine patients with drug-refractory epilepsy during visual stimulation with faces and mosaics. We analyzed event-related potentials (ERP), gamma frequency power, phase-amplitude coupling and phase-slope-index and compared the results between patients with versus without a mesial temporal SOZ.

**Results:**

In the amygdala and fusiform gyrus, faces triggered higher ERP amplitudes compared to mosaics in both patient groups and higher gamma power in patients without a mesial temporal SOZ. In the hippocampus, famous faces triggered higher gamma power for both groups combined but did not affect ERPs in either group. The differentiated ERP response to famous faces in the parahippocampal gyrus was more pronounced in patients without a mesial temporal SOZ. Phase-amplitude coupling and phase-slope-index results yielded bidirectional modulation between amygdala and fusiform gyrus, and predominately unidirectional modulation between parahippocampal gyrus and hippocampus.

**Conclusions:**

A mesial temporal SOZ was associated with an impaired response to faces in the amygdala, fusiform gyrus and parahippocampal gyrus in our patients. Compared to this, the response to faces in the hippocampus was impaired in patients with, as well as without, a mesial temporal SOZ. Our results support existing evidence for face processing deficits in patients with a mesial temporal SOZ and suggest the pathological effect of a mesial temporal SOZ on the amygdala to play a pivotal role in this matter in particular.

## Background

A mesial temporal seizure onset zone (SOZ) in temporal lobe epilepsy (TLE) has consistently been associated with deficits in social cognition [1–4]. One of the most basic levels of social cognition is constituted by the ability to recognize a face as a face, e.g. as a prerequisite to recognize and perceive face emotions [5]. Typically, the evaluation of this ability involves measuring face-selective responses in a distributed network of brain areas thought to be involved in face processing summarized as the “face network” [6]. Inside the face network, the brain area most strongly associated with face emotion recognition is the amygdala [7–9]. While the amygdala has a long history of being linked with face emotion recognition, its response to neutral faces compared to non-face stimuli has been gaining attention in the more recent years as well. Some studies showed the amygdala’s face-selectivity to be on a similar scale as, or sometimes even exceeding, the fusiform face area [10–12]. This face-selective response in the amygdala was suggested to be a key component in the amygdala’s contribution to face processing [13].

In connectivity analyses, the amygdala and hippocampus form a distinct cluster associated with emotional processes, memory formation and face discrimination [14, 15]. The hippocampus not only responds to faces [11, 12, 16] but also shows a differentiated response to familiar versus non-familiar faces [17, 18] and emotional faces [19]. From a pathological standpoint, the epileptogenic hippocampus represents one of the most common causes for focal epilepsy [20]. Inside the face network, evidence exists for the close connectivity between the amygdala and hippocampus to the fusiform and parahippocampal gyrus respectively [21–24]. The fusiform gyrus with its functionally localized fusiform face area [25] was suggested to receive direct influence from the amygdala in fearful face processing [26], and a face-specific connectivity between them was shown using neutral face stimuli [11]. The role of the parahippocampal gyrus was proposed to be that of ‘contextual processing’, meaning it provides the hippocampus with the information as to “where” and “when” an input was associated with [27]. Multiple studies have shown activation of both the hippocampus and parahippocampal gyrus during associative tasks involving faces (for review, see Ref. [28]).

While intracranially implanted electrodes ensure a high level of spatial and temporal precision, their use remains a feature in a fraction of all EEG studies in humans due to the invasive implantation procedure. Two established methods to analyze intracranial EEG consist of averaging the post-stimulus epochs to compute event-related potentials (ERP) and conducting a time–frequency analysis [18, 29–31]. In time–frequency analyses inside the face network, gamma frequencies have been studied most extensively out of all frequency bands [32, 33]. It has been proposed that synchronization in the gamma frequencies affect communication between neuronal groups and thus represent a fundamental process in neuronal connections subserving higher cognitive functions [34]. A number of studies compared ERPs and gamma power elicited by faces in selective sites of the visual network [35–38]. The two methods show different response characteristics to faces, and it has been speculated that gamma power is associated with elaborate processing of faces, while ERPs may represent synchronization within regions of the face processing network [39]. So far it is unclear if and how a SOZ in the examined area affects ERPs and gamma frequencies differently. Most intracranial EEG studies aim to examine healthy brain functionality and involve patients with a SOZ outside of their regions of interest only [40–42]. To our knowledge, no study examining face processing in the temporal lobe has compared epileptogenic versus non-epileptogenic functionality using intracranial EEG.

It was therefore the goal of our study to evaluate the effect of a SOZ on the EEG response to faces inside the temporal lobe. To accomplish this, intracranial ERPs and gamma power were measured in response to faces versus mosaics, and between different categories of faces, in the amygdala, hippocampus, fusiform and parahippocampal gyrus in patients with drug-refractory epilepsy. Different categories of neutral faces, including famous public figures and wooden masks as an abstract representation of faces, were chosen to test for the robustness of the response to faces. A linear mixed-effects model was implemented to compare the results between patients with a mesial temporal SOZ, Group M, and patients without a mesial temporal SOZ, Group O. As fMRI studies examining face processing in patients with a mesial temporal SOZ demonstrated decreased activity in the amygdala, hippocampus and parahippocampal gyrus on the side of seizure onset [43–45] and decreased functional connectivity in face-processing regions of the temporal lobe centered around the amygdala [46], we hypothesized to see a reduced ERP and gamma frequency response to faces in the examined regions of Group M compared to Group O.

In a second step, we analyzed the functional connectivity between the amygdala, hippocampus, fusiform gyrus, and parahippocampal gyrus. It has been proposed that gamma frequencies contribute to communication between neuronal groups through cross-frequency-coupling with lower frequency bands [34]. Out of all cross-frequency-couplings, phase-amplitude coupling (PAC) has gained interest in particular [15, 47–49]. This is due to the assumption that lower frequency phase modulates the time window in which neuronal spiking activity increases, which is then reflected by gamma frequency amplitude accordingly [50, 51]. Recent research suggests that when PAC is calculated between inter-regional intracranial electrodes, gamma frequency amplitude as an output of the higher order, “driver” area will be related to the low frequency phase of the lower order, “receiver” area [52]. Following this line of thinking, we hypothesized to find significant PAC between gamma frequency amplitude in the amygdala and low frequency (3–20 Hz) phase in the fusiform gyrus, and between gamma frequency amplitude in the hippocampus and low frequency phase in the parahippocampal gyrus. For further analysis of the modulation direction, we calculated the phase-slope-index (PSI), which also considers the time lag that is bound to occur when a signal modulates another in a remote area [53]. As our PSI calculations represent cumulative directionality over a span of 750 ms, we hypothesized to see bidirectional connectivity between the amygdala and fusiform gyrus as reported before using fMRI and Granger causality [54], as well as between the hippocampus and parahippocampal gyrus.

## Materials and methods

### Patients

**Fig. 1:**
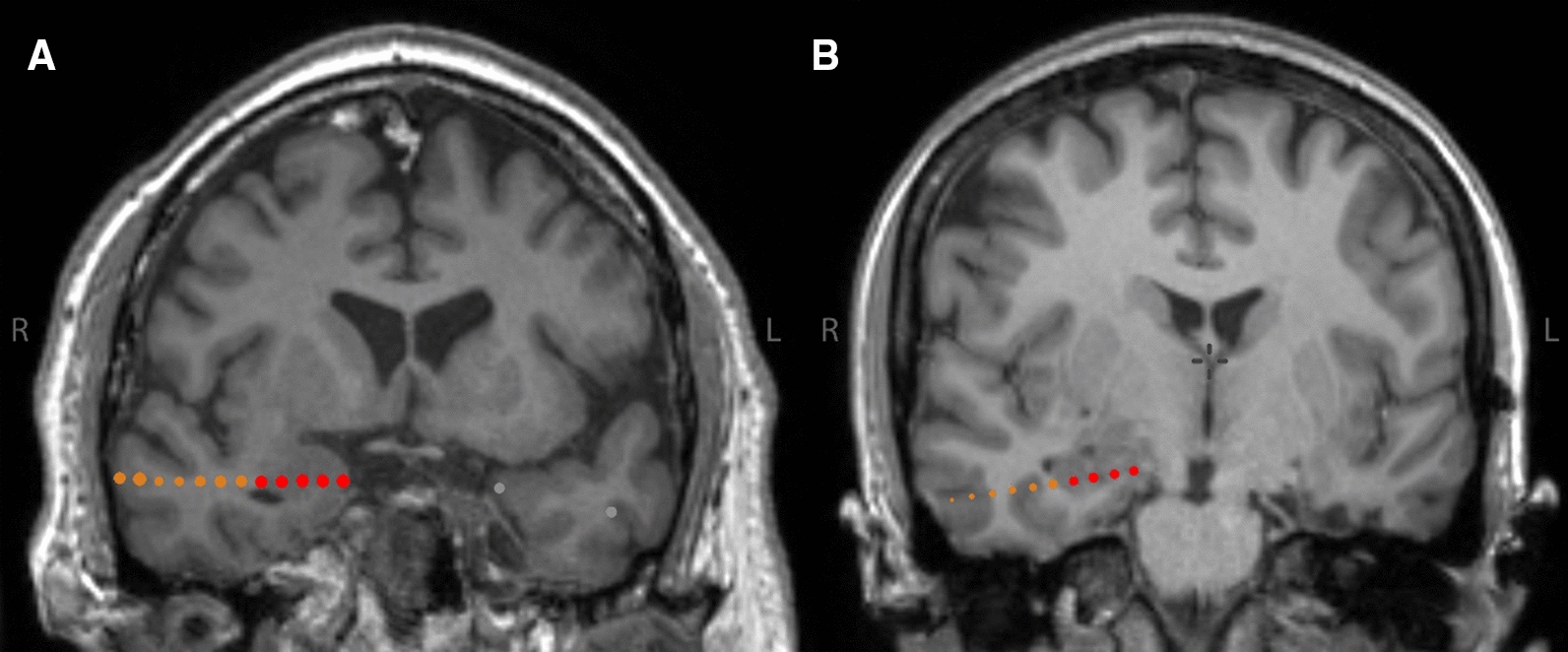
Examples of intracranial electrode localizations. Coronal MRI images (T1-weighted) of two patients. Red dots: Electrode contacts inside target structure. Orange dots: Electrode contacts outside target structure. Only data derived from electrode contacts inside target structure were analyzed. **A** Amygdala electrode contacts in patient 5, right hemisphere (Group M). **B** Hippocampus electrode contacts in patient 6, right hemisphere (Group O)

Nine patients with drug-refractory epilepsy participated in this study (aged 18 to 54 years, 5 female and 4 male), all of whom were stereotactically implanted with intracranial electrodes for pre-surgical diagnostics independent of this study (see Fig. 1 for examples of electrode localizations). Using intracranial EEG data, patients were divided into two groups according to their SOZ (Table 1). Group O (patients 1 to 4) included patients with an extratemporal SOZ, and Group M (patients 5 to 9) with a mesial temporal SOZ. For bilaterally implanted patients presenting a unilateral SOZ, the unaffected contralateral hemisphere was attributed to Group O (patients 5 and 6). All patients gave their written informed consent to participate in this study. The study protocol was approved by the institutional review board.

**Table 1:** Patients and clinical data

ID	Age	Sex	Hemisph	Electrode contacts	Seizure onset zone	Etiology	Seizure types
Group O							
1	27	F	R	AM- 4 FU-4 PH-3	Ipsilateral frontal	Postcontusional	BTCS
2	40	M	L	AM- 5 HI- 1 FU- 2	Ipsilateral frontal	Unknown	BTCS
3	52	F	R	AM- 2 HI- 3 PH- 1	Contralateral anterior temporal	FCD	Sensory, hyperkinetic
4	18	M	L	AM- 4 HI- 2 FU- 2	Contralateral insula	Unknown	Sensory, automatism, hyperkinetic, autonomic, BTCS
5	54	M	L	AM- 3 FU- 1 PH- 1	Contralateral	–	FIAS + automatism, BTCS
6	24	F	R	AM- 4 HI- 4 FU- 2 PH- 3	Contralateral	–	Emotional, automatism, FIAS, BTCS
Group M							
5	54	M	R	AM- 5 FU- 2 PH- 2	Mesial temporal	FCD + HS	FIAS + automatism, BTCS
6	24	F	L	FU- 4	Mesial temporal and neocortical temporal	FCD + HS	Emotional, automatism, FIAS, BTCS
7	40	F	L	AM- 4 HI- 2 FU- 3 PH- 3	Mesial temporal	FCD	FIAS + automatism, BTCS
8	29	F	L	AM- 3 HI- 2 FU- 4 PH- 6	Mesial temporal	HS	Autonomic, FIAS, BTCS
9	32	M	L	AM- 4 HI- 3 PH- 2	Mesial temporal	Unknown	Sensory, BTCS
9	32	M	R	PH- 1 HI- 3	Mesial temporal	Unknown	Sensory, BTCS

*AM* Amygdala, *FU* Fusiform gyrus, *HI* Hippocampus, *PH* Parahippocampal gyrus, *FCD* Focal cortical dysplasia, *HS* Hippocampal sclerosis, *FIAS* Focal impaired awareness seizure, *BTCS* Bilateral tonic–clonic seizure

### Experiment procedure

We chose facial portraits in the categories Caucasian faces, dark-skinned faces, faces of famous public figures, veiled faces, and wooden masks of African or oceanic decent, each containing 50 pictures, respectively. Caucasian faces and faces of famous public figures each contained 25 female and 25 male models, while dark-skinned and veiled faces comprised of 50 female models. Caucasian, dark-skinned and veiled faces did not contain any person familiar to the participating study patients. We added wooden masks as a category to evaluate the response to abstract representations of faces. Veiled faces were included to test for differences between whole faces and faces where the mouth and nose regions are hidden. Famous public figures were chosen out of an online database ranking the most famous public figures in each age group [55]. In all pictures, facial expressions range from neutral to smiling, with none of the pictures showing an obvious sign of negative emotion. Additionally, a non-face category of 50 colored mosaics was compiled, bringing the total sum of pictures to 300. All pictures were rated by 11 participants in a separate rating procedure according to valence and arousal using the self-assessment manikin [56], to ensure that the pictures are emotionally neutral. Valence was rated on a scale from 1 for unpleasant to 5 for pleasant and resulted in a mean value of 2.64 with a standard deviation of 0.80. Arousal was rated on a scale from 1 for calm to 5 for excited and resulted in a mean value of 2.83 with a standard deviation of 0.71.

During the visual stimulation experiment, patients were seated in a darkened room, facing a 66 × 44 cm monitor with a face-screen distance of 1 m. The interstimulus interval varied randomly between 2800 and 3000 ms, after which a black screen with a central white cross was shown for 300 ms as a visual focus. Each picture was scaled to 600 × 800 pixels and shown for 1500 ms. Patients were asked to passively view the images.

### EEG recording

Electrode localizations were determined using co-registered (Compumedics® Neuroscan™, CURRY 7) pre-implantation 3D-MRI-datasets (3-Tesla, T1-weighted) and post-implantation CT scans. Subsequently each contact was attributed to a respective anatomical structure according to an atlas of anatomy [57]. Only electrode contacts implanted into one of our four regions of interest were selected for data analysis: Amygdala, hippocampus, fusiform gyrus and parahippocampal gyrus.

EEG recordings were sampled at 1024 or 2048 Hz. All EEG data and statistical analyses were carried out using Matlab R2011a. Data sampled at 2048 Hz was down-sampled to 1024 Hz.

### Event-related potentials

**Fig. 2:**
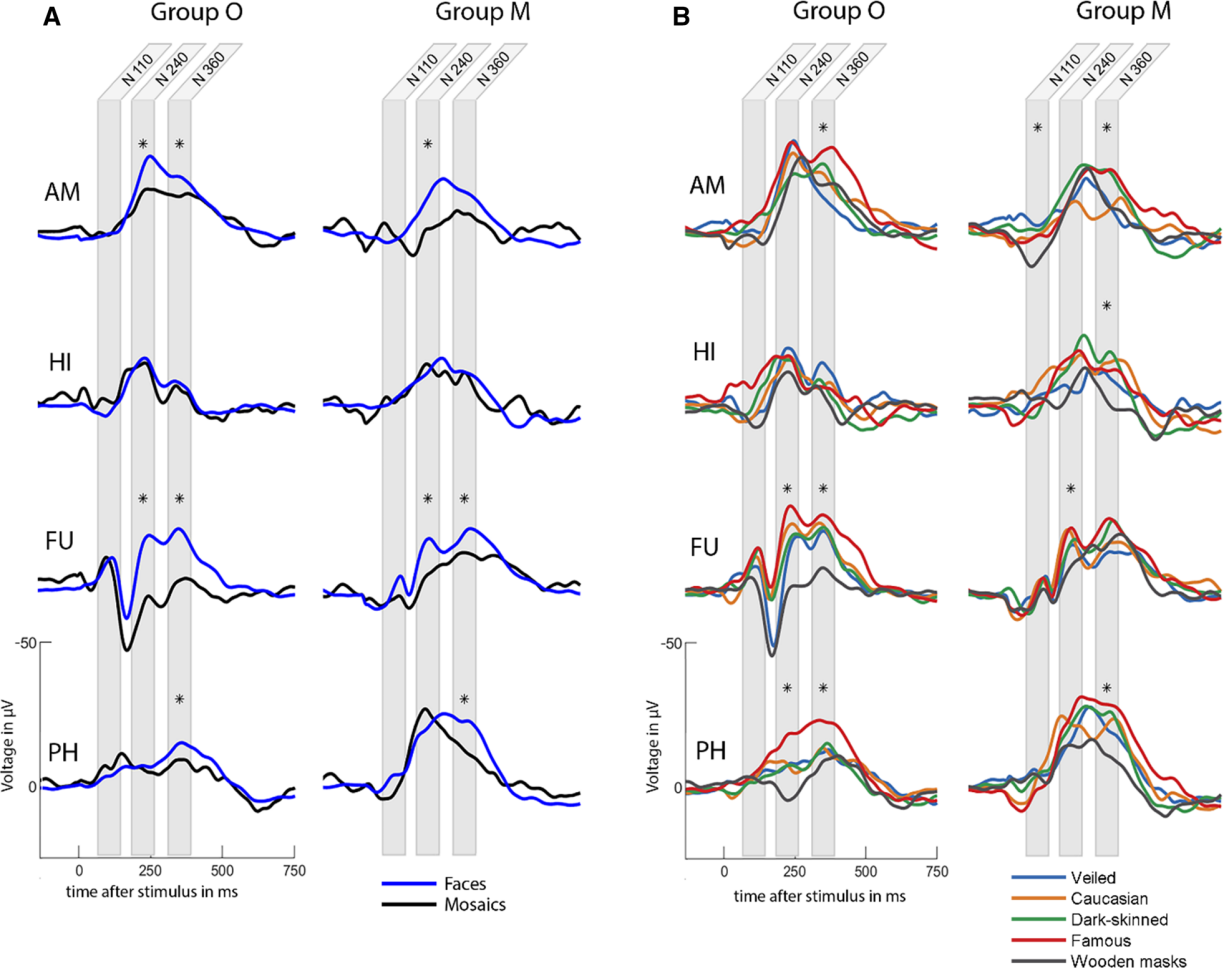
Event-related potentials. Grey areas mark the windows in which ERP amplitude was measured. Windows containing a significant categorical difference are marked with an asterisk (*). **A** ERPs for faces versus mosaics. In AM, N240 was higher for faces than mosaics in both groups, while N360 was higher only in Group O. Both groups showed higher N240 for faces in FU and higher N360 for faces in FU and PH. **B** ERPs for all face categories. In AM of Group O, N360 was higher for famous faces than wooden masks and veiled faces. In AM of Group M, N360 was higher for famous and dark-skinned faces than Caucasian faces, while N110 was lower for wooden masks than dark-skinned or veiled faces. In HI of Group M, N360 was lower for wooden masks than dark-skinned faces. In FU, wooden masks triggered lower amplitudes than all other face categories for N240 in both groups and N360 in Group O. In PH, N240 was higher for famous faces than dark-skinned faces and wooden masks in Group O. N360 was higher for famous faces than all other face categories in Group O and higher than wooden masks, Caucasian and veiled faces in Group M

ERPs were computed by averaging 1500 ms post-stimulus epochs and subtracting the mean of a pre-stimulus baseline of 1000 ms. Surface electrode Fz was used as the reference. A bandpass-filter between 0.5 and 20 Hz was applied. To remove outliers, epochs with a standard deviation greater than 2.5 times mean standard deviation across all epochs were rejected. This removal of outliers was performed for each electrode contact in each patient. Overall, the mean number of epochs that went into the statistical analyses per patient per electrode localization per category was 82 with a mean standard deviation of 13.1. Expected peak locations were defined to be around 110, 240 and 360 ms after stimulus onset based on visual inspection of the computed ERPs and information from published studies with a study design similar to ours: We mainly followed the ERP categorization of Barbeau et al. [18], who conducted intracranial ERPs during face recognition in multiple areas inside the temporal lobe including three out of four of our own regions of interest. This study identified an early ERP component peaking at 110 ms after visual stimulation in the fusiform gyrus, followed by stages of widespread parallel processing in different areas of the visual network at 240 and 360 ms post stimulus. In a different study, intracranial ERPs in the amygdala elicited a first negative peak at around 250 ms when viewing faces [36]. We hypothesize this peak to be comparable to the N240 described by Barbeau et al. [18]. For each of the above-mentioned peak locations, mean ERP amplitude was calculated in a window of 80 ms, which were marked in grey in Fig. 2. All graphs were plotted to show the ERP pointing upwards for easier viewing, i.e., multiplied by -1. All amplitudes mentioned below were adjusted accordingly.

### Gamma frequency analysis

**Fig. 3:**
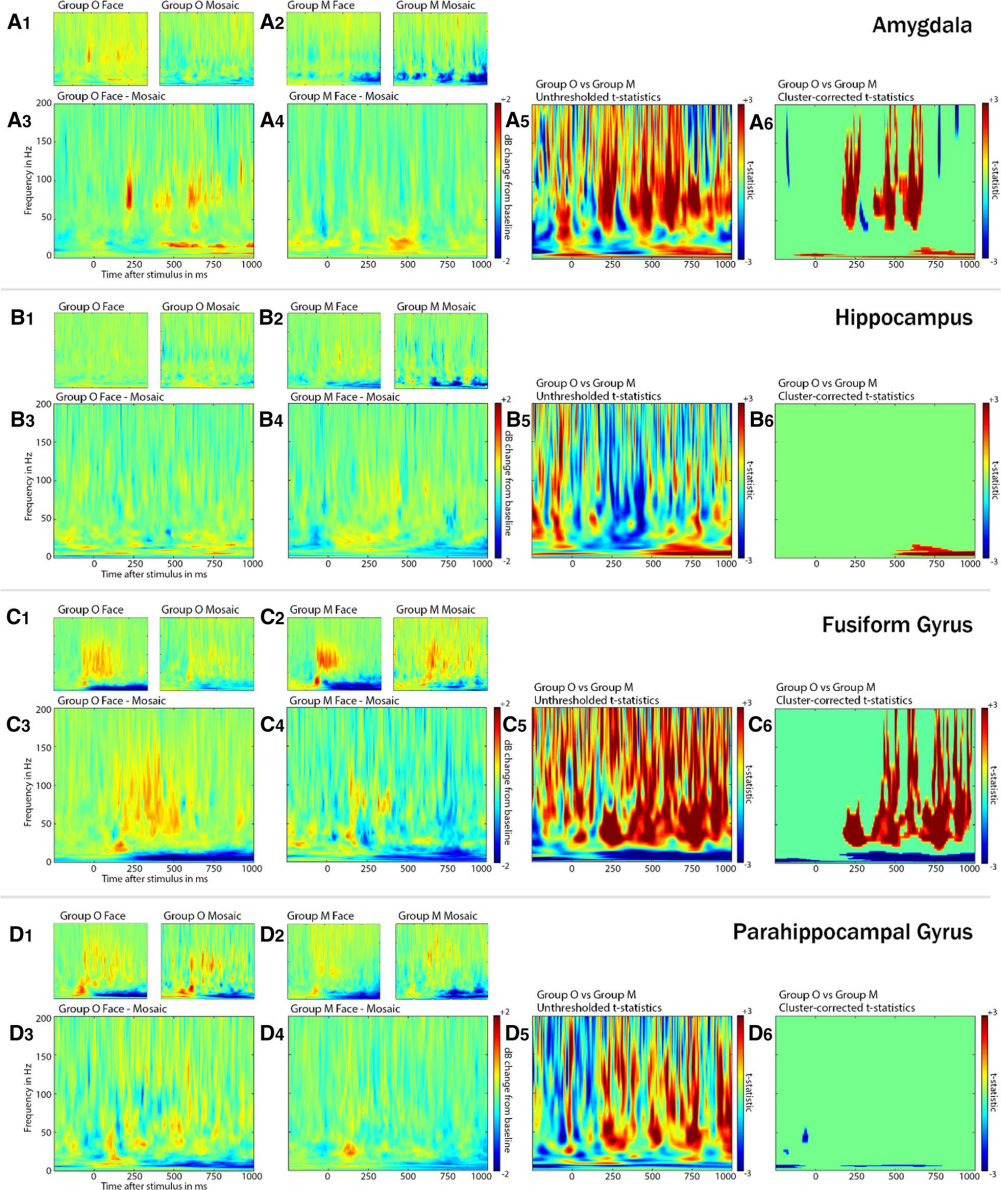
Gamma frequency analysis. **A**_**1**_–**D**_**1**_**, A**_**2**_–**D**_**2**_ Power map in response to faces and mosaics in Group O and Group M. Mean gamma power was measured during 0–750 ms in AM, FU and PH and 250–1000 ms in HI after stimulus onset and applied to the linear mixed-effects model. Gamma power was higher for faces than mosaics in AM (A1) and FU (C1) of Group O. **A**_**3**_–**D**_**3**_, **A**_**4**_–**D**_**4**_ Power map showing the response to faces minus mosaics in Group O and Group M. **A**_**5**_–**D**_**5**_ Unthresholded t-statistics comparing the power map for faces minus mosaics in Group O and Group M. Positive t-statistics indicate higher power in Group O and negative t-statistics indicate higher power in Group M. **A**_**6**_–**D**_**6**_ Remaining clusters of t-statistics after voxel-based and cluster-based thresholding at p ≤ 0.05. Large clusters in the gamma frequency range remained in AM and FU, indicating higher gamma power in Group O

Gamma frequency analysis was computed using a bipolar reference. The same method of artefact rejection using standard deviation as in the ERP calculation was applied here as well. We subtracted the ERP from each epoch to remove any effect that is phase-locked to the ERP. A time–frequency representation was computed using fast Fourier transform and wavelet convolution with complex Morlet wavelets in 40 logarithmically increasing frequencies between 1 and 200 Hz. Baseline normalization was performed via conversion into decibel using a pre-stimulus period of 1100 ms. We then extracted the mean power between 45 and 150 Hz during 1–750 ms (early-onset gamma) and 250 – 1000 ms (late-onset gamma) and applied the linear mixed-effects model. In order to find significant clusters of gamma power, we first subtracted the time–frequency representation for mosaics from that of faces (Fig. 3 A3–D3, A4–D4). We then compared the face minus mosaic time–frequency representation between Group O and Group M by performing a two-tailed two-sample t-test for each pixel of the time–frequency representation to create a map of test statistic values (Fig. 3A5–D5). Then a permutation test was performed by randomly dividing all patients into two groups and computing the same aforementioned two-sample *t*-test, which was repeated for 3000 times. In each permuted map of t-statistics, as well as in the real map of t-statistics, all voxels beneath a threshold corresponding to a p-value of 0.05 were removed. The sum of t-statistics in each remaining cluster was calculated and the maximum and minimum sum of each permuted map registered. We determined the 95th percentile from the distribution of maximum values and the 5^th^ percentile from the distribution of minimum values. In the real map of t-statistics, all clusters with a sum of t-statistics above the 5th and beneath the 95th percentile were removed (Fig. 3A6–D6) [58, 59].

### Linear Mixed-Effects Models and Statistical Analyses

All statistical analyses were carried out using R. Using the lme4 package [60], we built two linear mixed-effects models with ERP amplitude defined as the dependent variable and differing fixed effects. The fixed effects for Model 1 were group (O and M), picture category (face and mosaic), hemisphere (left and right), peaks (N110, N240 and N360) and the interaction between group and picture category. For each of the four electrode localizations, all trials by all patients conducted in the respective electrode localization were pooled together and entered into Model 1. The fixed effects for Model 2 were group, all face categories (Caucasian, dark-skinned, famous, veiled and masks), hemisphere, peaks and the interaction between group and face categories. As we were only interested in the face categories in Model 2, we removed all trials containing the mosaics category from the pooled trials and entered them into this model. Both models included patient as a random effect to account for non-independence of trials and were fit by restricted maximum likelihood. Visual inspection of residual plots was used to ensure that assumptions on linearity, homogeneity of variance and normality of residuals were met. P-values were calculated using the lmerTest package [61], which applies Satterthwaite’s method to generate p-values. If the effect of the interaction between group and category was significant, we followed up with post hoc pairwise comparisons using the emmeans package [62] with built-in Tukey method to adjust for multiple comparisons. For each electrode localization and each peak, we also conducted pairwise comparisons for picture or face category using the emmeans package. To evaluate gamma power, we used both Model 1 and 2 without peaks as a fixed effect. All p-values excluding those already adjusted in the post hoc analyses were corrected for multiple comparisons using the Holm-Bonferroni method. The corrected p-values were considered significant at p < 0.05. Confidence intervals reported under Results were not corrected for multiple comparisons.

### Phase-amplitude coupling and phase-slope-index

PAC between low frequency phase (3–20 Hz) and gamma frequency amplitude (45–200 Hz) was computed for a chosen range of electrode pairs between (1) the amygdala and fusiform gyrus, (2) the hippocampus and parahippocampal gyrus, and (3) the amygdala and hippocampus. Eight out of nine patients were implanted in both amygdala and fusiform gyrus, six patients in both hippocampus and parahippocampal gyrus, and seven patients in both amygdala and hippocampus. For each of these patients, we visually inspected the time frequency representation of every electrode inside the aforementioned structures, to choose one electrode with the least amount of artifacts for each structure. Inter-regional PAC was then calculated for each patient’s electrode pair by multiplying the gamma frequency amplitude time series of one structure with the low frequency (3–20 Hz) phase time series of the other, and vice versa, over 750 ms after stimulus presentation [50]. PAC results were converted into Z-scores and considered significant at z ≥ 2.

Following this, we computed PSI for all electrode pairs showing a significant amount of PAC. PSI was calculated between low frequency phase and gamma power envelope using cross frequency coherence and a segment length of 750 ms [63]. Only the PSI results within the phase and amplitude range revealed as significant according to each electrode pair’s respective PAC results were considered. All PSI results were converted into Z-scores and considered significant at z ≥ 2 or ≤ − 2.

## Results

For an overview of the statistical results for ERP and gamma frequency analysis, see Tables 2 and 3.

**Table 2:** Linear mixed-effects model results for ERP and gamma frequency analysis

	ERP			Gamma		
	β	95% CI	p-value	β	95% CI	p-value
AM						
1						
Group	2.27	[− 3.70, 8.24]	ns	0.62	[0.07, 1.16]	ns
Hemisphere	2.42	[− 3.13, 7.97]	ns	0.09	[− 0.30, 0.49]	ns
Faces vs mosaics	6.4	[4.47, 8.32]	0.001	− 0.02	[− 0.34, 0.30]	ns
Group * F vs M	–	–	ns	–	–	0.027
Peaks	–	–	< 0.001			
2						
Group	− 1.60	− 7.78, 4.59	ns	− 0.04	− 0.68, 0.60	ns
Hemisphere	2.84	− 2.90, 8.59	ns	0.05	− 0.41, 0.50	ns
Face categories	–	–	< 0.001	–	–	ns
Group * face categories	–	–	< 0.001	–	–	ns
Peaks	–	–	< 0.001			
HI						
1						
Group	4.93	[− 6.13, 16.00]	ns	0.18	[− 0.02, 0.37]	ns
Hemisphere	6.39	[3.38, 9.39]	0.003	− 0.12	[− 0.38, − 0.03]	ns
Faces vs mosaics	3.99	[− 2.82, 10.80]	ns	− 0.12	[− 0.44, 0.19]	ns
Group * F vs M	–	–	ns	–	–	ns
Peaks	–	–	< 0.001			
2						
Group	− 5.00	[− 17.89, 7.89]	ns	0.32	[− 0.13, 0−77]	ns
Hemisphere	6.07	[2.92, 9.22]	0.015	− 0.12	[− 0.31, 0.08]	ns
Face categories	–	–	0.012	–	–	0.013
Group * face categories	–	–	ns	–	–	ns
Peaks	–	–	< 0.001			
FU						
1						
Group	− 0.07	[− 2.43, 2.31]	ns	− 0.33	[− 0.73, 0.09]	ns
Hemisphere	1.40	[− 0.71, 3.50]	ns	− 0.25	[− 0.65, 0.16]	ns
Faces vs mosaics	10.3	[8.71, 12]	< 0.001	0.90	[0.45, 1.34]	0.007
Group * F vs M	–	–	< 0.001	–	–	ns
Peaks	–	–	< 0.001			
2						
Group	− 2.91	[− 6.57, 0.75]	ns	− 0.06	[− 0.45, 0.32]	ns
Hemisphere	1.40	[− 0.78, 3.57]	ns	− 0.07	[− 0.37, 0.23]	ns
Face categories	–	–	< 0.001	–	–	ns
Group * face categories	–	–	< 0.001	–	–	ns
Peaks	–	–	< 0.001			
PH						
1						
Group	21.1	[16.7, 25.5]	< 0.001	0.26	[− 0.29, 0.81]	ns
Hemisphere	27.86	[24.26, 31.45]	< 0.001	− 0.32	[− 0.71, 0.07]	ns
Faces vs mosaics	4.7	[3.1, 6.29]	< 0.001	− 0.14	[− 0.62, 0.34]	ns
Group * F vs M	–	–	ns	–	–	ns
Peaks	–	–	< 0.001			
2						
Group	14.6	[10.1, 19.1]	< 0.001	− 0.09	[− 0.61, 0.79]	ns
Hemisphere	23.12	[19.32, 26.91]	< 0.001	− 0.28	[− 0.70, 0.14]	ns
Face categories	–	–	< 0.001	–	–	ns
Group * face categories	–	–	ns	–	–	ns
Peaks	–	–	< 0.001			

Asterisk represents that an interaction between two variables was examined

*AM* Amygdala, *FU* Fusiform gyrus, *HI* Hippocampus, *PH* Parahippocampal gyrus, *ns* Not significant (p ≥ 0.05)

No β-values and confidence intervals are reported for p-values that were followed up by post-hoc analysis

**Table 3:** Linear mixed effects model post-hoc analysis for ERP peaks

	Group O			Group M		
	β	95% CI	p-value	95% CI	95% CI	p-value
AM						
1						
N110 faces vs mosaics	0	[− 6.52, 6.53]	ns	− 0.13	[− 7.32, 7.06]	ns
N240 faces vs mosaics	10.04	[3.51, 16.56]	< 0.001	14.28	[7.09, 21.46]	< 0.001
N360 faces vs mosaics	8.49	[1.97, 15.02]	0.003	5.86	[− 1.33, 13.05]	ns
2						
N110 dark- skinned vs masks	3.02	[− 6.51, 12.55]	ns	11.14	[0.67, 21.61]	0.024
N110 veiled vs masks	− 0.90	[− 10.24, 8.44]	ns	13.99	[3.16, 24.81]	0.001
N360 famous vs masks	9.66	[0.25, 19.08]	0.038	8.33	[− 1.98, 18.63]	ns
N360 famous vs veiled	15.15	[5.43, 24.88]	< 0.001	9.39	[− 1.13, 19.92]	ns
N360 famous vs Caucasian	4.33	[− 4.61, 13.26]	ns	14.25	[3.04, 25.46]	< 0.001
N360 dark- skinned vs Caucasian	− 2.18	[− 11.24, 6.88]	ns	15.48	[4.13, 26.83]	< 0.001
HI						
1						
N110 faces vs mosaics	− 3.87	[− 12.04, 4.30]	ns	− 0.87	[− 9.48, 7.74]	ns
N240 faces vs mosaics	2.64	[− 5.53, 10.81]	ns	− 0.92	[− 9.53, 7.69]	ns
N360 faces vs mosaics	3.68	[− 4.49, 11.85]	ns	1.82	[− 6.79, 10.43]	ns
2						
N360 dark- skinned vs masks	2.11	[− 10.80, 15.03]	ns	15.85	[3.17, 28.52]	0.002
FU						
1						
N110 faces vs mosaics	1.65	[− 5.55, 8.85]	ns	2.69	[− 1.92, 7.29]	ns
N240 faces vs mosaics	22.25	[15.05, 29.45]	< 0.001	8.93	[4.32, 13.53]	< 0.001
N360 faces vs mosaics	19.41	[12.21, 26.61]	< 0.001	7.13	[2.53, 11.74]	< 0.001
2						
N240 famous vs masks	27.47	[16.89, 38.05]	< 0.001	11.57	[4.70, 18.45]	< 0.001
N240 dark- skinned vs masks	18.69	[7.88, 29.50]	< 0.001	8.94	[2.03, 15.85]	0.001
N240 veiled vs masks	14.94	[4.35, 25.53]	< 0.001	7.25	[0.18, 14.31]	0.038
N240 Caucasian vs masks	25.64	[15.78, 35.51]	< 0.001	10.47	[3.01, 17.94]	< 0.001
N360 famous vs masks	16.64	[6.06, 27.22]	< 0.001	10.73	[− 1.63, 23.10]	ns
N360 dark- skinned vs masks	13.98	[3.17, 24.79]	0.001	12.19	[− 1.34, 25.72]	ns
N360 veiled vs masks	17.42	[6.83, 28.02]	< 0.001	7.81	[− 4.84, 20.46]	ns
N360 Caucasian vs masks	19.34	[9.47, 29.20]	< 0.001	4.35	[− 2.55, 11.25]	ns
PH						
1						
N110 faces vs mosaics	− 3.38	[− 9.13, 2.37]	ns	6.34	[0.92, 11.77]	0.011
N240 faces vs mosaics	3.4	[− 2.35, 9.15]	ns	2.68	[− 2.75, 8.10]	ns
N360 faces vs mosaics	6.57	[0.82, 12.32]	0.014	12.47	[7.04, 17.90]	< .0001
2						
N240 famous vs dark- skinned	8.98	[0.84, 17.12]	< 0.001	0.45	[− 7.66, 8.55]	ns
N240 famous vs masks	15.74	[7.56, 23.91]	< 0.001	7.62	[− 0.46, 15.71]	ns
N360 famous vs dark- skinned	8.17	[0.03, 16.31]	0.048	2.87	[− 5.24, 10.97]	ns
N360 famous vs masks	15.59	[7.42, 23.76]	< 0.001	13.31	[5.23, 21.40]	< 0.001
N360 famous vs veiled	9.55	[1.20, 17.89]	0.009	9.51	[1.26, 17.76]	< 0.001
N360 famous vs Caucasian	10.12	[2.29, 17.95]	0.001	9.70	[1.16, 18.24]	< 0.001

*AM* Amygdala, *FU* Fusiform gyrus, *HI* Hippocampus, *PH* Parahippocampal gyrus, *ns* Not significant (p ≥ 0.05)

In Model 2, post-hoc analyses with non-significant p-values in both Group O and Group M were omitted from the table for viewing purposes

### Responsive versus non-responsive electrode contacts

Each of the 51 electrode contacts in Group O and 53 electrode contacts in Group M were examined individually. In Group O, 40 (78%) electrode contacts had an ERP in response to faces, 27 (53%) had enhanced gamma power in response to faces, and 26 (51%) had both ERPs and gamma power. In Group M, ERP was seen in 44 (83%), gamma power in 23 (43%), and both ERPs and gamma power in 22 (42%) electrode contacts in response to faces. Combining both groups together, two (4%) out of 50 electrode contacts with face-induced gamma power did not show a face-induced ERP, while 36 (43%) out of 84 electrode contacts with face-induced ERP did not show face-induced gamma power.

### Response to faces versus mosaics

In the amygdala, faces triggered higher ERP amplitudes than mosaics, which was visible in N240 in both groups and N360 in Group O only. For gamma amplitude, the linear mixed-effects model did not show an effect of picture category for both groups combined, but the interaction between group and picture category was significant. To study this interaction effect, pairwise comparisons were performed, which revealed faces to trigger higher gamma power compared to mosaics in Group O (β = 0.70, 95% CI [0.44, 0.96], p = 0.017; Fig. 3A1) but not in Group M (β = − 0.11, 95% CI [− 0.45, 0.23], p > 0.05; Fig. 3A2). The gamma cluster analysis confirmed this result by displaying large clusters in the gamma frequency range with positive test statistic values indicating higher gamma power in Group O than Group M (Fig. 3A6). The earliest face-induced gamma cluster was shown to be around 200 ms after stimulus onset. These results combined demonstrate that, while both groups show a differentiated response to faces in the amygdala, Group O’s response is stronger on a statistically significant level. From the interaction effect between group and picture category, which was significant in the linear mixed-effects model for gamma power but not ERPs, we can also conclude that this difference between Group O and Group M was more pronounced in the gamma analysis.

The fusiform gyrus exhibited the most prominent difference between faces and mosaics out of all examined regions. ERP amplitude was higher in response to faces in N240 and N360 in both groups. While the linear mixed-effects model yielded an interaction effect between group and picture category, pairwise comparisons showed faces to trigger higher ERPs than mosaics in Group O (β = 14.4, 95% CI [11.6, 17.3], p < 0.001) as well as Group M (β = 6.25, 95% CI [4.42, 8.08], p < 0.001). Comparing the estimated marginal means for faces and mosaics in each group thus revealed that the interaction effect stems from Group O showing an even higher difference between faces and mosaics than Group M. The linear mixed-effects model for gamma power analysis also displayed a difference between faces and mosaics for both groups combined. An interaction effect between group and picture category could not be shown after correcting for multiple comparisons. As the p-value was significant before applying the Holm-Bonferroni method, pairwise comparisons were still performed. These resulted in higher gamma power in response to faces in Group O (β = 0.90, 95% CI [0.30, 1.49], p < 0.001; Fig. 3C1) but not in Group M (β = − 0.05, 95% CI [− 0.59, 0.49], p > 0.05; Fig. 3C2). In accordance to this, our gamma cluster analysis demonstrated higher gamma power in Group O than Group M, which lasted until almost 1000 ms after stimulus onset (Fig. 3C6).

In the hippocampus, neither ERP amplitude, nor gamma power could be shown to give rise to a distinct response to faces compared to mosaics, with no differences between Group O and Group M either. In the parahippocampal gyrus, N360 amplitude was higher for faces in both groups, while gamma power analysis did not reveal a difference between faces and mosaics in either group. Gamma cluster analysis also did not display any significant clusters comparing faces and mosaics in neither the hippocampus (Fig. 3B6), nor the parahippocampal gyrus (Fig. 3D6). But visual inspection of the gamma power plots presented an important difference between the hippocampus and parahippocampal gyrus. While gamma power in the hippocampus was almost non-existent for both picture categories and groups (Fig. 3B1, B2), the parahippocampal gyrus displayed a noticeable gamma response to both faces and mosaics in both groups (Fig. 3D1, D2). The fact that the response to mosaics was just as pronounced as the response to faces in the parahippocampal gyrus, led to the lack of a measurable difference between the two stimuli there.

### Face categories

Conclusively, the linear mixed-effects models revealed ERP amplitudes to differ between face categories in each examined region. When we studied the category differences across all regions, two face categories stood out. Firstly, famous faces were recorded to trigger higher ERPs compared to other categories most often, i.e., N240 in the fusiform gyrus of both groups, N360 in the amygdala and parahippocampal gyrus of both groups, N240 in the parahippocampal gyrus of Group O, and N360 in the fusiform gyrus of Group O. While the hippocampus was the only region where this distinct ERP response to famous faces was not measured, it was the only region that did show a difference between face categories in the gamma analysis. Pairwise comparisons revealed gamma power in the hippocampus to be highest for famous faces and significantly higher compared to dark-skinned faces (β = 0.52, 95% CI [0.06, 0.98], p = 0.017), wooden masks (β = 0.49, 95% CI [0.05, 0.92], p = 0.022) and veiled faces (β = 0.43, 95% CI [0.01, 0.85], p = 0.049). There was no interaction effect between group and face categories, meaning that this distinct reaction to famous faces can be attributed to both groups. Secondly, wooden masks triggered lower ERPs more often than other categories, i.e., N240 in the fusiform gyrus of both groups, N360 in the parahippocampal gyrus of both groups, N360 in the fusiform gyrus of Group O, N240 in the parahippocampal gyrus of Group O, N360 in the hippocampus of Group M, and N110 in the amygdala of Group M. Gamma power, on the other hand, was not shown to be lower for wooden masks compared to other categories.

### PAC and PSI

**Fig. 4:**
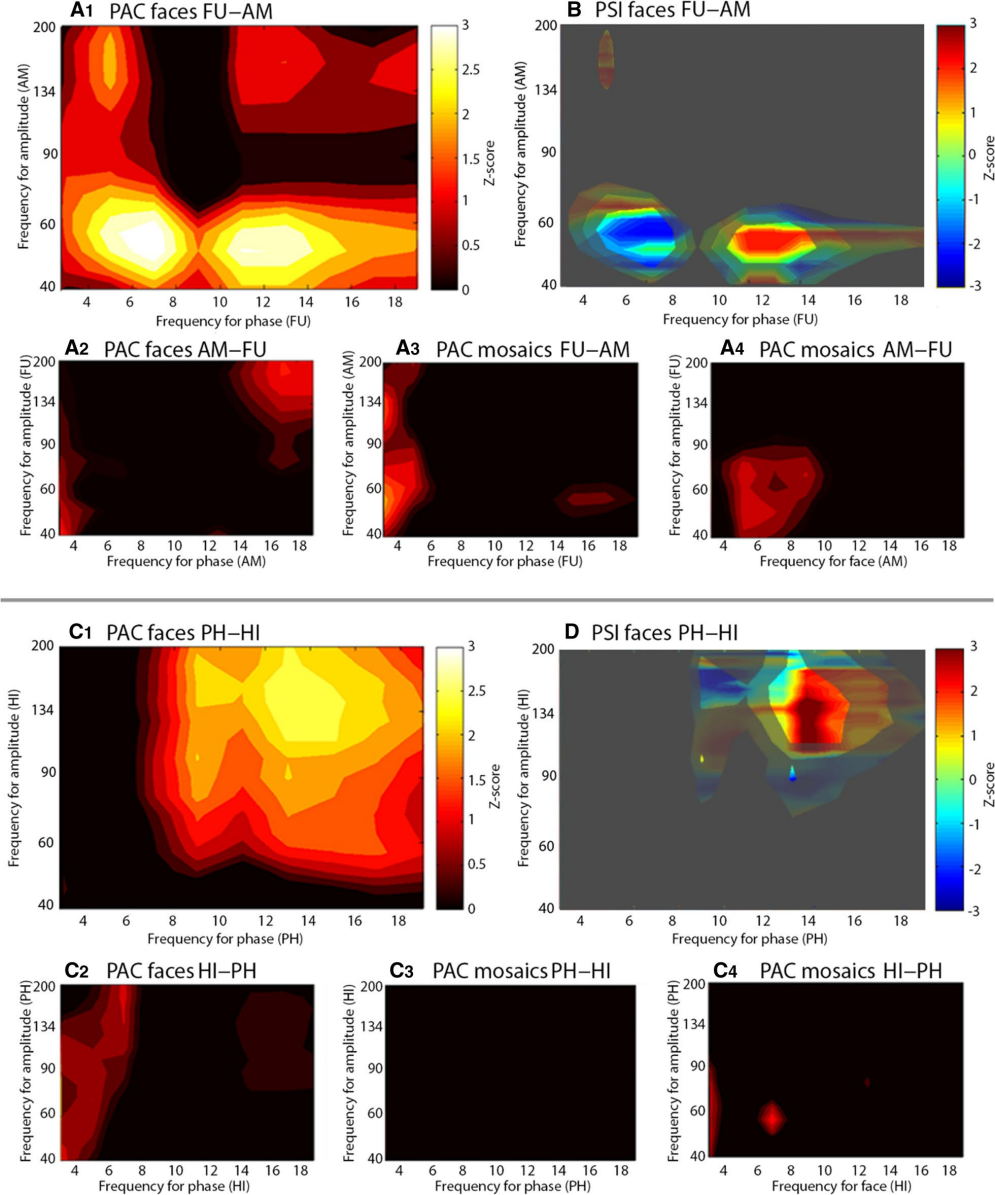
Phase-amplitude coupling and phase-slope index. **A1**–**A4** PAC between AM and FU in patient 6, right hemisphere (Group O). Enhanced PAC (Z-score ≥ 2) was observed between FU theta/alpha phase and AM low gamma frequency amplitude when viewing faces. No enhanced PAC was observed when viewing mosaics. **B** Overlap between PSI and the area of enhanced PAC. Red area indicates modulation directionality from FU theta phase towards AM low gamma amplitude. Blue area indicates modulation directionality from AM low gamma amplitude towards FU alpha phase. **C1–4** PAC between HI and PH in patient 9, right hemisphere (Group M). Enhanced PAC (Z-score ≥ 2) was calculated between PH alpha/beta phase and HI high gamma amplitude when viewing faces. No enhanced PAC was observed when viewing mosaics. **D** Overlap between PSI and the area of enhanced PAC. Red area indicates modulation directionality from PH alpha/beta phase towards HI high gamma amplitude. Blue areas indicate modulation directionality from HI high gamma amplitude towards PH alpha phase

Between the amygdala and the fusiform gyrus, three out of eight electrode pairs showed PAC between low frequency phase in the fusiform gyrus and gamma amplitude in the amygdala, while showing no PAC in the reverse constellation (i.e. low frequency phase in the amygdala and gamma amplitude in the fusiform gyrus; see Fig. 4A1–4 for an example). One out of eight electrode pairs showed PAC in both of the aforementioned constellations.

Between the hippocampus and the parahippocampal gyrus, three out of six electrode pairs showed PAC between low frequency phase in the parahippocampal gyrus and gamma amplitude in the hippocampus, with no coupling in the reverse constellation (i.e., low frequency phase in the hippocampus and gamma amplitude in the parahippocampal gyrus) (see Fig. 4C1–4 for an example).

Between the amygdala and hippocampus, one out of seven electrode pairs showed PAC between low frequency phase in the amygdala and gamma amplitude in the hippocampus, with no coupling in the reverse constellation.

The remaining electrode pairs showed no enhanced PAC.

Overall, eight out of nine significant PAC computations resulted in considerably smaller to non-existent couplings for mosaics compared to faces.

PSI between low frequency phase in the fusiform gyrus and gamma amplitude in the amygdala was calculated for four electrode pairs, with phase and amplitude range specified according to each pair’s respective PAC. All four electrode pairs showed both positive and negative PSI values, indicating bidirectional modulation between low frequency phase in the fusiform gyrus and gamma amplitude in the amygdala (see Fig. 4B for an example).

Between low frequency phase in the parahippocampal gyrus and gamma amplitude in the hippocampus, PSI was calculated for three electrode pairs. While all three pairs revealed negative PSI values, only one of them additionally showed a positive PSI value as well (see Fig. 4D for an example). Negative PSI values indicate modulation directionality from gamma amplitude in the hippocampus towards low frequency phase in the parahippocampal gyrus, while positive PSI values indicate the opposite direction.

PSI between low frequency phase in the amygdala and gamma amplitude in the hippocampus was calculated for the single electrode pair showing enhanced PAC and revealed both positive and negative values, indicating bidirectional modulation.

## Discussion

### ERP and gamma frequency analysis

We set out to compare the response to faces in specific regions of the temporal lobe between patients with and without a mesial temporal SOZ. Conclusively, we saw predominantly preserved, non-epileptogenic functionality in the amygdala, fusiform and parahippocampal gyrus in patients without a mesial temporal SOZ (Group O), while patients with a mesial temporal SOZ (Group M) displayed impaired functionality in all of the examined regions, which we will illustrate in the following.

Previous studies examining non-epileptogenic amygdala functionality have shown faces to trigger a negative intracranial ERP peak comparable to N240 and a subsequent lower peak comparable to N360 [8, 36], as well as intracranial gamma power activity starting from 200 ms after stimulus onset in the amygdala [36]. Compared to this, our amygdala results can be summarized as follows: While Group O presented both higher N240 and N360 for faces, as well as the first face-induced gamma cluster around 200 ms, Group M was only able to replicate a higher N240 for faces compared to mosaics. Sato et al. [36] speculated this response to faces starting from 200 ms in the amygdala to be related to conscious perceptual processing of faces, referencing the “visual awareness negativity”, a prominent ERP peak observed around 200–300 ms in scalp EEG for consciously detected visual stimuli only [64, 65]. As Group O was able to replicate non-epileptogenic amygdala functionality while Group M was not, we interpret from these results that conscious perception of faces may be impaired in patients with a mesial temporal SOZ. Multiple behavioral studies examining humans and primates associate impaired amygdala functionality with impaired perception of faces: It was reported that monkeys with bilateral amygdala lesions had no viewing preference for faces or face-like objects and also exhibited changed face-viewing behavior, i.e. no advantage for the eyes or mouth region [66]. This lack of fixation on the eye region was also seen in a human patient with bilateral amygdala damage [67]. In a patient with unilateral amygdala damage, gaze shift towards the eye and mouth regions was impaired in brief (150 ms) stimulus presentations, while it was normal during longer (5000 ms) presentations [68]. These results indicate that the amygdala is involved in the rapid detection of facial features, which in turn could explain why patients with a mesial temporal SOZ were reported to score lower than healthy controls in tests involving the recognition of face emotions and familiar faces [4, 69, 70]. Studies on unfamiliar face recognition on the other hand revealed conflicting results [71, 72], demonstrating the necessity for conducting further electrophysiological studies.

The hippocampal ERPs of our patients, while presenting visible N240 and N360 peaks, did not show a distinction between faces and mosaics. Although Halgren et al. [73] in their intracranial EEG study recorded a face-specific N240 in a portion of their hippocampal electrode contacts and a non-specific N360, they and also Barbeau et al. [18] described a later positive peak, P480, to be the dominant ERP component in the hippocampus. Contrary to this, visual inspection of our own ERPs implies that P480 was not a prominent factor in the hippocampi of our patients. When individual face categories were compared, none of the ERP peaks showed a distinction to famous faces either, contrary to results from studies examining non-epileptogenic hippocampal functionality [18, 30].

In the fusiform gyrus, ERPs altogether closely replicated the sequence of N110-P160-N240 defined by Barbeau et al. [18], which is also comparable to the sequence of N130-P180-N240 in the study by Halgren et al. [73]. Both studies not only observed the N110-P160-N240 in the fusiform gyrus in response to faces but attributed the role of the principal generator of the sequence to the fusiform gyrus, exerting a causal influence on other regions of the temporal lobe. In particular, they postulated the N110 to represent a rapid feed-forward signal triggering further processing: Halgren et al. [73] theorized the N110-P160-N240 to represent a bottom-up sequence with the fusiform gyrus receiving a “primitive sketch” from the lower-level visual cortices during N110 and projecting face-specific encoding to higher cortical areas during P160 and N240. P160 possibly corresponds to N170, a well-studied ERP component that multiple source localization studies reported to originate from face-selective regions in the fusiform gyrus and occipitotemporal cortex (for review, see [74]). While N170 or P160 was repeatedly demonstrated to be larger in response to faces compared to non-face stimuli [75, 76], P160 in the fusiform gyrus of our patients revealed lower amplitudes for faces than mosaics in both groups, for which the reason is unclear. In our gamma analysis, both groups exhibited early face-induced gamma power (Fig. 3C1, C2), but the gamma cluster analysis indicated higher gamma power for Group O that lasted until > 500 ms after stimulus onset (Fig. 3C6), similar to results from studies exploring healthy fusiform gyrus functionality [77, 78].

ERPs in the parahippocampal gyrus, while not replicating the clear triphasic N240-P300-N360 observed by Barbeau et al. [18], did show higher N360 in response to faces than mosaics in both groups. Interestingly, the parahippocampal gyrus in Group O showed the most distinguished ERP response to famous faces out of all regions examined. Studies using fMRI and MEG have shown the parahippocampal cortex to be associated with extracting familiar contextual associations from visual stimuli [79], with an increase in phase synchrony between the parahippocampal cortex and other regions of the contextual associations network starting at 150–250 ms after stimulus onset [80]. Combined with these insights, our results suggest that the parahippocampal N240 and N360 are associated with the presence of contextual information of a face. Furthermore, our connectivity analyses detected PAC between low frequency phase in the parahippocampal gyrus and gamma frequency amplitude in the hippocampus, while a directed modulation of the hippocampus by the parahippocampal gyrus could not be shown. This reflects a feedforward projection from the parahippocampal gyrus to the hippocampus during face processing and also suggests that the source of the impaired response to famous faces seem to be rooted in the hippocampus, as opposed to the parahippocampal gyrus.

Thus, we can conclude that a mesial temporal SOZ was associated with an impaired ERP and gamma power response to faces in the amygdala, fusiform gyrus and parahippocampal gyrus in our patients. While this part of our hypothesis was confirmed, we did not expect both our patient groups to display an impaired response to faces in the hippocampus. In a similar fashion, Halgren et al. [73] noted that a large number of their hippocampal contacts did not show distinct ERPs and the ones who did displayed extremely variable waveforms, while amygdala contacts generally showed clear ERPs even in patients with no hippocampal ERPs. They attributed the lack of hippocampal ERPs to structural damage arising from hippocampal sclerosis. When we break down the patients in Group O, one third of the patients had a SOZ ipsilateral extratemporal, and another third in the contralateral mesial temporal lobe in the form of hippocampal sclerosis. Evidence for structural damage in the hippocampus exists for both of these etiologies: In patients with extratemporal epilepsy with normal hippocampal MRI, magnetic resonance spectroscopic imaging could demonstrate the presence of structural damage in the hippocampus [81], while previously T2 relaxometry had revealed abnormalities [82]. Evidence also exists for structural anomalies in the contralateral hippocampus at the presence of unilateral mesial-TLE [83, 84]. A possible explanation for the involvement of the contralateral hippocampus is seizure propagation through the dorsal hippocampal commissure [85, 86], although the functional relevance of this structure is disputed [87, 88]. These findings could explain why hippocampal EEG responses were similarly impaired in both groups of our study.

A limitation of our study is the fact that two patients are incorporated in both patient groups, as the hemisphere containing the SOZ was attributed to Group M and the unaffected hemisphere to Group O. Our linear mixed-effects model accounts for this by assigning a different random intercept for each patient in addition to the fixed effects of group and hemisphere. This way the non-independence of observations is taken into consideration. Nevertheless, the limited number of patients prevented us from being able to make group comparisons for PAC and PSI results. Further research involving a higher number of patients is necessary for better assessment of the pathological effect of a SOZ on the connectivity of specific temporal lobe regions during face processing.

### PAC and PSI

Due to the restricted number of electrode pairs that we examined for our PAC and PSI calculations, we refrained from making comparative statements between Group O and M. We expected to find significant PAC between gamma frequency amplitude in the amygdala and low frequency phase in the fusiform gyrus, and between gamma frequency amplitude in the hippocampus and low frequency phase in the parahippocampal gyrus. This turned out to be the case in the majority of the examined electrode pairs, indicating feedback modulation from the amygdala to the fusiform gyrus, and from the hippocampus to the parahippocampal gyrus. Keeping the interpretational differences between local and inter-regional PAC in mind, our PAC results are still in accordance with the theory of “predictive coding” [89], which says that brain areas simultaneously communicate through lower and higher frequencies representing feedback predictions and feedforward prediction errors. Our results indicate that the amygdala and the hippocampus represent “driver” areas that send feedback predictions to the fusiform gyrus and the parahippocampal gyrus, which represent “receiver” areas and in turn send feedforward prediction errors.

The PSI results in the hippocampus and parahippocampal gyrus predominately indicate unidirectional modulation of the parahippocampal gyrus by the hippocampus. In the amygdala and the fusiform gyrus, however, PSI results indicate bidirectional modulation in all examined electrode pairs. Previously, bidirectional connectivity between the amygdala and fusiform gyrus was shown using fMRI and Granger analysis [54] as well as dynamic causal modeling [23] during visual processing. Considering our PSI results represent cumulative results over 750 ms, a strictly unidirectional modulation seems less plausible than bidirectional. Therefore, this discrepancy possibly represents further evidence for impaired hippocampal functionality in our patients.

## Conclusions

In summary, a mesial temporal SOZ was associated with an impaired ERP and gamma power response to faces in the amygdala, fusiform gyrus and parahippocampal gyrus in our patients. Compared to this, the response to faces in the hippocampus was impaired in patients with, as well as without, a mesial temporal SOZ. Our connectivity analyses showed bidirectional modulation between the amygdala and fusiform gyrus when viewing faces, and predominately unidirectional modulation between the hippocampus and the parahippocampal gyrus, which further suggests overall higher levels of functional impairment in the hippocampi compared to the amygdalae in our patients. Our results support existing fMRI and behavioral studies reporting deficits in face processing in patients with a mesial temporal SOZ, with the added benefit of the spatial and temporal accuracy of intracranial EEG. Our results further suggest the pathological effect of a mesial temporal SOZ on the amygdala to play a pivotal role in this matter in particular.

## Data Availability

The raw datasets analyzed for the current study are not publicly available because we did not obtain the consent of participants to provide them to third parties for publication, but they are available from the corresponding author on reasonable request.

## Abbreviations

AM: Amygdala
ERP: Event-related potential
FU: Fusiform gyrus
HI: Hippocampus
PAC: Phase-amplitude coupling
PH: Parahippocampal gyrus
PSI: Phase-slope-index
SOZ: Seizure onset zone
TLE: Temporal lobe epilepsy
